# Utilization of Tomato (*Solanum lycopersicum*) Peel Powder as a Flavor Enhancer and Natural Antioxidant in Sausage Formulation

**DOI:** 10.1155/ijfo/9998340

**Published:** 2025-12-12

**Authors:** Xuyuan Nie, Meina Jiang, Kai Wang, Gang Kuang, Jing Hu, Qiang Wang, Yan Li

**Affiliations:** ^1^ School of Biological and Chemical Engineering, Chongqing University of Education, Chongqing, China, cqu.edu.cn; ^2^ College of Food Science and Nutritional Engineering, China Agricultural University, Beijing, China, cau.edu.cn; ^3^ Scientific Research Department, Chongqing Medical and Pharmaceutical College, Chongqing, China, cqmu.edu.cn

**Keywords:** DPPH scavenging activity, lipid oxidation, sausages, sensory properties, tomato peel powder

## Abstract

This research was aimed at investigating the potential of utilizing antioxidant‐rich tomato peels, an underutilized byproduct in agroindustry, for enhancing the quality and nutritional attributes of sausages. The study incorporated varying concentrations and particle sizes (0.425 mm coarse/RD vs. 0.18 mm fine/RE) of tomato peel powder into sausage formulations, followed by an assessment of their sensory characteristics and antioxidant capacity. Results indicated that the inclusion of tomato peel powder led to a decrease in brightness (*L* ^∗^) while augmenting redness (*a* ^∗^) and yellowness (*b* ^∗^) parameters compared to the control group. Moreover, it positively influenced the flavor profile of the sausages, with overall acceptability scores surpassing 7 across all test groups containing tomato peel powder (though the control group scored 4.13 in aroma). The study also revealed that tomato peels had a retarding or inhibitory effect on lipid oxidation within the sausages, with coarse particles (0.425 mm) showing significantly stronger suppression than fine particles (0.18 mm). Throughout a 24‐day refrigerated storage period, TBARSs (thiobarbituric acid reactive substances) and POV (peroxide value) measurements were notably slower in the treatment groups, particularly RD20 (20% starch substitution with 1.0% tomato peel powder) and RD40 (40% starch substitution with 2.0% tomato peel powder), as opposed to the untreated samples. Notably, RD10 (10% starch substitution with 0.5% tomato peel powder) exhibited the longest oxidation induction period (8.2 h vs. RD20′s 7.3 h). DPPH radical scavenging activity assays showed that sausage formulations with 20% starch substitution (1.0% tomato peel powder) exhibited stronger antioxidant capacity than those with 0.01% butylated hydroxytoluene (BHT). Finally, the use of coarse particles of tomato peel enhanced the fatty acid profile by improving PUFA retention; for instance, the RD10 treatment showed a 12.3% increase compared to RE10, thereby improving the sausages′ nutritional quality. This study underscores the feasibility and benefits of valorizing tomato peels with optimized particle size and substitution ratio in sausage production for improved product quality and sustainability.

## 1. Introduction

Sausages, being a widely enjoyed meat product, are nonetheless subject to oxidative degradation, which negatively impacts their sensory attributes—namely, flavor, texture, and hue. To mitigate this issue, numerous food additives, such as butylated hydroxyanisole (BHA) and butylated hydroxytoluene (BHT) [1], have been integrated into sausage formulations to regulate lipid oxidation. However, consumer preferences increasingly favor products without these synthetic antioxidants due to potential health risks, as they have been linked to carcinogenicity [1, 2]. Consequently, there is an urgent need to explore alternatives to synthetic antioxidant usage in sausage products.

Tomato is a widely consumed global vegetable, with an annual world production capacity of 40 million tons [3]. This seasonal crop, aside from its fresh consumption, is predominantly processed into various other forms such as paste, juice, and puree [4]. Consequently, the worldwide generation of tomato byproducts, inclusive of peel, pulp, and seeds, is estimated to be 5.4 × 10^6^–9.0 × 10^6^ tonnes [5]. However, these tomato pomaces are commonly disposed of as waste or utilized for animal feed [6], leading to challenges in waste management and environmental pollution.

In this context, it is crucial to address the issue of tomato pomace utilization, given its substantial contribution to agricultural waste streams and the potential negative environmental impact. The valorization of these byproducts through innovative processing techniques could not only mitigate disposal problems but also contribute to sustainable resource management and economic efficiency within the food industry.

Outshining the seeds and pulp, tomato peel—a currently underutilized agroindustrial byproduct—offers a superior nutritional profile: high dietary fiber (84.16%, primarily 71.82% insoluble hemicellulose), alongside significant phenolics (e.g., rutin and naringenin), ascorbic acid, and lycopene (3–4 mg/100 g) [6]. The dietary fiber in tomato peel residue is a good water‐holding agent, and lycopene is a natural antioxidant and natural pigment [7, 8]. Adding tomato peel residue to processed foods can increase the fiber content, thereby improving the texture, water retention, and taste of foods; improve stability and extend shelf life; and improve color, which can enhance appetite [9–11]. This suggests that the judicious exploitation of tomato peel as a valuable resource, particularly for its natural antioxidant properties, can facilitate its valorization while concurrently mitigating environmental impact. One pragmatic approach involves incorporating the peel directly into food products, which may provide a more cost‐effective solution than isolation procedures [12].

Tomato peel powder (TPP) has been shown to enhance the nutritional and functional properties of sausages. Studies demonstrated that the incorporation of 10% airflow ultramicro crushed TPP reduced saturated fatty acid content by 18% and increased the polyunsaturated/saturated fatty acid ratio (P/S) by 23% compared to control sausages, aligning with the WHO recommendations for cardiovascular health [13]. Additionally, it was highlighted that its rich nondigestible fiber (84.16% total dietary fiber) and lycopene (3.2 mg/100 g), which contribute to gut microbiota modulation and oxidative stress reduction, are key markers of metabolic health [6, 14]. Currently, direct comparisons between TPP and synthetic antioxidants (e.g., BHT) under standardized storage conditions remain underexplored. The present study addresses these gaps by evaluating two particle sizes (0.425 and 0.18 mm) and multiple substitution levels (10%, 20%, and 40%) of TPP as a starch substitute, aiming to clarify how granulometry influences antioxidant capacity, sensory properties, and lipid oxidation kinetics. This granularity‐specific analysis is critical for maximizing the functional potential of tomato peel in meat products, as particle size can affect bioactive compound release, texture, and color development—factors that directly impact consumer acceptance and product shelf life.

This study developed a novel sausage formulation enriched with TPP, specifically exploring its use as a starch substitute and the effect of particle size on product quality. The research uniquely evaluated the natural antioxidant capacity of the powder by direct comparison with synthetic antioxidants under standardized storage, in addition to assessing the sensory attributes, to validate its potential for creating healthier, clean‐label foods.

## 2. Materials and Methods

### 2.1. Materials

Fresh pork loin (lean meat content ≥ 90%, fat content ≤ 8%) and back fat (subcutaneous fat, thickness 2–3 cm) were obtained from a local supermarket (Yonghui, Chongqing, China) within 24 h postslaughter and stored at 4°C before use. Tomato peel was provided by Xinghua Lvbao Food Co. Ltd. (Jiangsu, China) and dried by hot air. Tomato powder was made by a mill (Chongqing Instrument Manufacturing Co. Ltd., Chongqing, China) at 3000 rpm for 1 min. The rough TPPs were then sieved through 40‐mesh (aperture 0.425 mm) and 80‐mesh (aperture 0.180 mm) stainless steel sieves (GB/T 6003.1‐2012 standard) to obtain TPP with particle sizes < 0.425 and < 0.180 mm, respectively. A total antioxidant capacity (T‐AOC) assay kit was obtained from Shanghai Macklin Biochemical Technology Co. Ltd., Shanghai, China.

### 2.2. Sausage Preparation

The sausage production process adhered to the previously documented protocols [13] with modifications as follows: A total of eight sausage formulations were fabricated according to the raw material composition detailed in Table 1. TPP (particle sizes: 0.425 and 0.18 mm) was used as a starch substitute at four inclusion levels: 0%, 10%, 20%, and 40% (*w*/*w*). A control group was included with 0.01% food‐grade BHT (Macklin, CAS No. 128‐37‐0) instead of TPP.

**Table 1:** Sausage formulation with different levels of tomato peel powder.

**Sausage formulas**	**Starch substitution (%)**	**Materials in meat mixture (g/kg)**				
		**Pork back fat**	**Lean pork**	**Tomato peel powder**	**Corn starch**	**Compound starch**
B	0	300	700	0	60	140
C	0	300	700	0	60	140
RD10	10	300	700	6	54	140
RD20	20	300	700	12	48	140
RD40	40	300	700	24	36	140
RE10	10	300	700	6	54	140
RE20	20	300	700	12	48	140
RE40	40	300	700	24	36	140

Abbreviations: B, blank control; C, sausage with 0.01% BHT (positive control); RD, tomato peel powder particle size 0.425 mm; RD10/RE10, 10% of the starch in the sausage is replaced by tomato peel powder; RD20/RE20, 20% of the starch in the sausage is replaced by tomato peel powder; RD40/RE40, 40% of the starch in the sausage is replaced by tomato peel powder; RE, tomato peel powder particle size 0.18 mm.

Pork meat was diced to a uniform 10‐mm particle size and mixed with all ingredients. After salting at 4°C for 2 h, the mixture was stuffed into 22 mm diameter composite casing using a sausage stuffer (Model 15L, Zhejiang Jihui Co. Ltd., China). Thermal processing was performed in a vertical retort (Model MDC‐DMZ‐GYJ‐6, Huadao Energy‐Saving Technology Co. Ltd., China) at 121°C (15 psi) for 30 min, followed by cooling to 25°C within 1 h. Samples were stored at 4°C. All experiments were conducted in triplicate with independent batches.

### 2.3. Color Evaluation

The coloration of sausage was quantitatively assessed using a HunterLab Colorimeter (HunterLab, Virginia, United States), an industry‐standard device. Prior to measurements, the colorimeter was calibrated against a certified white reference tile exhibiting *L* ^∗^ = 93.97, *a* ^∗^ = −0.08, and *b* ^∗^ = 1.21. The degree of coloration is represented by the values of lightness (*L* ^∗^), redness (*a* ^∗^), and yellowness (*b* ^∗^) parameters. Three sausages in each group were used to measure the colors.

### 2.4. Sensory Evaluation

In the sensory evaluation of the sausages, a panel consisting of eight seasoned experts was convened. Private booths under white fluorescent lights were prepared for each member as previously described [15]. The sausages were cooked in an oven and subsequently sliced into 3 cm thick portions before being randomized for presentation to each participant. A 5‐min interval was maintained between the provision of each sample, with water available for palate cleansing purposes. Each sausage attribute was assessed using a 1–10 point scale as follows: texture (ranging from 1, indicating *extreme dissatisfaction*, to 10, signifying *utmost satisfaction*), color (with 1 representing *extreme dislike* and 10 denoting *extreme satisfaction*), tenderness (where 1 equates to *extreme dislike* and 10 indicates *extreme satisfaction*), aroma (from 1 for *extreme dislike* to 10 for *extreme satisfaction*), and overall acceptability (spanning from a score of 1 for *extreme dislike* to 10 for the *highest level of satisfaction*).

### 2.5. Sample Preparation and T‐AOC Determination

For the determination of T‐AOC, approximately 1.000 g of TPP (sieved to 0.425 mm) was accurately weighed and transferred into a 50 mL centrifuge tube. Subsequently, 20.0 mL of 70% ethanol was added to the sample. The mixture was subjected to ultrasonic extraction under the following conditions: 200 W power, 40 kHz frequency, and 50°C for 30 min. After ultrasonic treatment, the mixture was centrifuged at 8000 rpm for 5 min. The supernatant was carefully transferred to a 50 mL volumetric flask and made up to volume with 70% ethanol. The resulting extract was then filtered through a 0.45 *μ*m membrane filter to ensure clarity and homogeneity. A stock solution of DPPH (2,2‐diphenyl‐1‐picrylhydrazyl) was prepared by dissolving an appropriate amount of DPPH powder in 70% ethanol to achieve an initial absorbance of approximately 1.0 at 515 nm. An aliquot of 20 *μ*L of the prepared extract was mixed with 80 *μ*L of DPPH working solution (final concentration of 0.025 mg/mL DPPH in methanol) in a 96‐well plate. The mixture was incubated at room temperature in the dark for 30 min to allow for the reaction between DPPH and the antioxidant compounds in the sample. After incubation, the absorbance was measured at 515 nm using a microplate reader. The absorbance of the blank (methanol instead of sample) was recorded as *A*_0_, and the absorbance of the sample was recorded as *A*_1_. The DPPH radical scavenging activity was calculated using the following formula:

DPPH Scavenging Activity %=1−A1AO×100%.

### 2.6. Determination of Thiobarbituric Acid Reactive Substance (TBARS) Values

The TBARS levels were quantified according to the procedures outlined in the National Standard of China [16]. To initiate the process, a 5‐g aliquot of the sample was subjected to homogenization in an oscillating apparatus for 30 min at 50°C, employing 50 mL of a trichloroacetic acid (TCA) solution fortified with 2.98 mM EDTA in 7.5% TCA (*w*/*v*). Subsequently, upon reaching ambient temperature, the homogenate was filtered through qualitative filter paper (diameter: *Φ*12.5 cm; pore size: 8–11 *μ*m; flow rate: medium). In the next step, 5 mL of the filtrate was combined with an equal volume of 0.02 M thiobarbituric acid (TBA), and the mixture was incubated in a water bath at 90°C for 30 min, followed by cooling to room temperature naturally. The absorbance of the resulting solution was measured at 532 nm using a spectrophotometer.

### 2.7. Determination of Peroxide Value (POV)

The POV was ascertained through an end‐point titration methodology in strict adherence to the National Standard of China [17]. The fat extraction from the sausage was executed by incorporating petroleum ether for an incubation period of 12 h. Subsequently, the sample was subjected to rotary evaporation to eliminate the petroleum ether residue, following which it was meticulously filtered using Whatman filter paper impregnated with sodium sulfate anhydrous. The filtrate was then treated with a 30 mL admixture of chloroform and glacial acetic acid, after which it was combined with a 0.5 mL aliquot of saturated potassium iodide solution, under dark conditions, allowing the reaction to proceed for 5 min. Ultimately, the resultant filtrate was subjected to titration against a standardized 0.01 M sodium thiosulfate solution. The POV was reported in milliequivalents per kilogram of sample.

### 2.8. Determination of Fatty Acid Profile and Induction Period

The fatty acid composition of a sausage oil methyl ester was ascertained according to the National Food Safety Standard [18] guidelines utilizing gas chromatography (Agilent 7890A model, manufactured in the United States). The oxidative stability assessment of the sausage lipid component was executed using a dedicated oil oxidation stability analyzer. The process involved an accelerated oxidation test at a temperature of 120°C and an airflow rate of 10 L/h. All analyses were conducted in triplicate to ensure statistical reliability.

### 2.9. Statistical Analysis

Data expressed as mean ± SD. Analyses used SPSS 26.0. Assumptions verified: normality via the Shapiro–Wilk test and variance homogeneity via Levene′s test, both with *p* > 0.05. One‐way ANOVA was conducted for group differences. If significant, Duncan′s test was used for pairwise comparisons. *p* < 0.05 is considered significant.

## 3. Results and Discussion

### 3.1. Color Properties

The color was analyzed via Duncan′s multiple range test (*p* < 0.05) with triplicate measurements per group (Table 2). Results revealed significant effects of TPP concentration and particle size on sausage color.

**Table 2:** Effect of different levels of tomato peel powder on sausage color values: Lightness (*L* ^∗^), redness (*a* ^∗^), and yellowness (*b* ^∗^).

**Sausage formulas**	**L** ^∗^	**a** ^∗^	**b** ^∗^
B	79.17 ± 0.83^a^	18.21 ± 0.79^c^	5.92 ± 0.34^d^
RD10	76.25 ± 0.64^b^	25.18 ± 4.38^b^	17.95 ± 0.42^c^
RD20	75.06 ± 0.74^c^	23.13 ± 0.55^b^	23.88 ± 1.59^b^
RD40	73.02 ± 0.28^d^	22.95 ± 0.58^d^	27.00 ± 2.10^b^
RE10	74.02 ± 1.15^d^	20.48 ± 0.54^e^	19.71 ± 1.18^c^
RE20	74.35 ± 0.95^d^	24.13 ± 0.83^b^	25.31 ± 0.65^b^
RE40	72.56 ± 0.21^e^	25.58 ± 0.29^a^	32.71 ± 0.49^a^

*Note:* Different lowercase letters in the same column indicate statistically significant differences among group mean values (*p* < 0.05), as determined by one‐way analysis of variance (ANOVA), followed by Duncan′s multiple range test.

For lightness (*L* ^∗^), the control group (B, 0% TPP) exhibited the highest value (79.17 ± 0.83^a^), while *L* ^∗^ decreased with increasing TPP concentration (*p* < 0.05). For coarse TPP (0.425 mm, RD groups), *L* ^∗^ values were 76.25 ± 0.64^b^ (RD10, 10% starch substitution), 75.06 ± 0.74^c^ (RD20, 20%), and 73.02 ± 0.28^b^ (RD40, 40%). In addition, fine TPP (0.18 mm, RE groups) showed lower *L* ^∗^ than RD groups at the same substitution level: 74.02 ± 1.15^d^ (RE10, 10%), 74.35 ± 0.95^d^ (RE20, 20%), and 72.56 ± 0.21^e^ (RE40, 40%). This reduction in *L* ^∗^ is attributed to the absorption of light by TPP′s natural pigments (e.g., lycopene and chlorophyll) and the dietary fiber matrix, consistent with Wang et al., who reported decreased in *L* ^∗^ in sausages with tomato peel addition due to pigment deposition, and Cadariu et al., who found that fine TPP has a lower *L* ^∗^ value than coarse power due to better pigment dispersion [12, 19].

Redness (*a* ^∗^) significantly increased with TPP inclusion compared to the control (B: 18.21 ± 0.79^c^). The highest *a* ^∗^ was observed in RE20 (25.58 ± 0.29^a^, 20% fine TPP), followed by RD10 (25.18 ± 4.38^b^, 5% coarse TPP) and RE20 (24.13 ± 0.83^b^, 10% fine TPP). This enhancement is likely due to lycopene, a carotenoid in tomato peel that imparts red coloration [19]. Notably, *a* ^∗^ values did not differ significantly between RD and RE groups at the same substitution level (*p* > 0.05), suggesting particle size has a minor impact on redness, which aligns with Cadariu et al., who found that lycopene release is primarily concentration‐dependent [12].

Yellowness (*b* ^∗^) also increased with TPP concentration (*p* < 0.05). The control (B) had the lowest *b* ^∗^ (5.92 ± 0.34^d^), while RE40 (32.71 ± 0.49^a^, 20% fine TPP) and RD40 (27.00 ± 2.10^b^, 20% coarse TPP) showed the highest values. This is attributed to carotenoids (e.g., *β*‐carotene) in tomato peel, which contribute to yellow hues [20]. Fine TPP (RE groups) generally had higher *b* ^∗^ than coarse TPP (RD groups) at the same substitution level (e.g., RE20: 25.31 ± 0.65^b^ vs. RD20: 23.88 ± 1.59^b^), possibly due to increased surface area enhancing carotenoid extraction [12]. Although high *b* ^∗^ values are sometimes associated with undesirable yellowing, sensory evaluation (Table 3) indicated all TPP‐added sausages had color scores ≥ 7.5, with RE20 and RE40 scoring 8.13 ± 1.10^a^, suggesting the reddish‐yellow hue was acceptable to panelists. Similar results have been reported by Kim et al. [19].

**Table 3:** Sensory scores of sausages formulated with different levels of tomato peel powder.

**Sausage formulas**	**Texture**	**Color**	**Aroma**	**Tenderness**	**Overall acceptability**
B	7.38 ± 1.49^a^	5.63 ± 1.70^a^	4.13 ± 1.29^b^	5.88 ± 1.66^a^	7.63 ± 1.41^a^
RD10	7.63 ± 1.56^a^	7.63 ± 1.24^a^	7.88 ± 1.10^a^	7.89 ± 0.87^a^	7.75 ± 1.68^a^
RD20	7.38 ± 1.41^a^	8.00 ± 0.94^a^	6.38 ± 1.41^ab^	7.13 ± 0.87^a^	7.13 ± 1.73^a^
RD40	7.88 ± 1.10^a^	7.50 ± 1.41^a^	6.75 ± 1.13^ab^	7.13 ± 1.37^a^	8.00 ± 1.15^a^
RE10	8.38 ± 0.66^a^	8.13 ± 1.10^a^	8.38 ± 0.66^a^	8.63 ± 0.46^a^	8.38 ± 0.66^a^
RE20	7.88 ± 1.20^a^	8.13 ± 1.10^a^	8.38 ± 0.66^a^	7.88 ± 1.29^a^	8.63 ± 0.66^a^
RE40	8.50 ± 0.67^a^	8.13 ± 0.57^a^	8.13 ± 0.74^a^	8.00 ± 0.94^a^	8.50 ± 0.67^a^

*Note:* Different letters in the same column indicate significant differences among groups for the individual variables based on Duncan′s test (*p* < 0.05).

### 3.2. Sensory Properties

Table 3 shows sensory scores for various attributes of sausages supplemented with TPP. All sausage samples were acceptable, with scores exceeding 7—a threshold representing *moderate acceptability*, where ≥ 7 indicates products are *sensorily acceptable* to consumers [19]. The sensory characteristics of sausages with TPP addition up to 1.5% (starch substitution) remained unchanged, consistent with Kim et al. [19]. In our study, color and tenderness scores of TPP‐supplemented sausages were comparable to or slightly higher than the blank control (B), likely due to TPP′s natural pigments and water‐holding capacity [13]. Notably, aroma scores differed significantly (*p* < 0.05) between treatments, with RE groups (0.18 mm particle size) outperforming RD groups (0.425 mm). For example, RE10 (10% starch substitution) achieved an aroma score of 8.38 ± 0.66, significantly higher than RD10 (7.88 ± 1.10) and control (4.13 ± 1.29). This improvement may stem from TPP′s bioactive compounds (e.g., phenolics and terpenes) and volatile aroma components, which enhance flavor complexity and mask lipid oxidation‐derived off‐odors [21]. Importantly, TPP addition at 10%, 20%, and 40% starch substitution did not negatively impact sensory properties but improved flavor, addressing concerns about high TPP content by demonstrating optimized particle size (0.18 mm) and substitution ratios (10%–20%) that mitigate potential textural issues [15].

### 3.3. T‐AOC Determination of Tomato Peel

**Figure 1:**
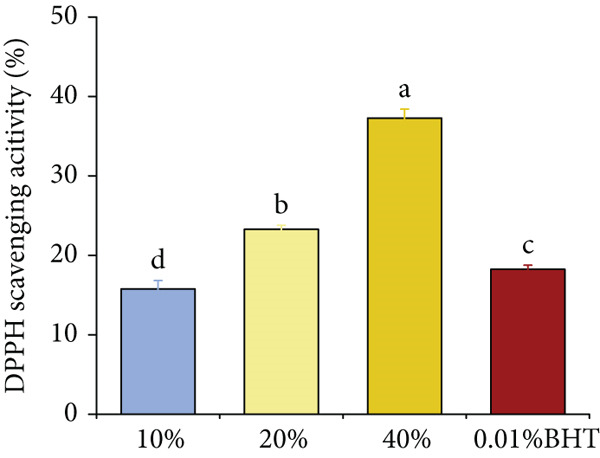
Antioxidant capacity of tomato peel. Different lowercase letters indicate significant differences among different treatment groups (*p* < 0.05).

The results (Figure 1) indicated that as the substitution level of starch with tomato peel in the sausage formulation was raised from 10% to 40%, the DPPH radical scavenging activity increased accordingly from 15.76% to 37.27%. This finding demonstrates a significant enhancement in the antioxidant capacity of the sausages, which was directly correlated with the increased proportion of tomato peel. For comparison, the sample containing 0.01% BHT (a synthetic antioxidant used as a positive control) exhibited a DPPH radical scavenging activity of 18.25%. This value was slightly higher than that of sausages with 10% tomato peel substitution but substantially lower than those with 20% and 40% substitution levels. The oxidative induction period is the lag time before significant lipid oxidation occurs under accelerated conditions (e.g., 120°C in this study). It directly reflects antioxidant efficacy: A longer period indicates stronger inhibition of oxidation initiation (e.g., free radical formation), with RD20 (longest period) demonstrating optimal antioxidant performance [22]. These findings suggest that, at an inclusion level of 20% or higher, tomato peel as a starch substitute can confer a more potent antioxidant effect in sausages than the addition of 0.01% BHT.

### 3.4. Effect of TPP on Lipid Oxidation Characteristics of Sausage TBARSs

The impact of lipid oxidation on sausage flavor was investigated by monitoring TBARS levels to assess the efficacy of TPP in mitigating this process (Figure 2). Over a 36‐day refrigerated storage period, all samples exhibited a general increase in TBARS values, reflecting progressive lipid peroxidation and malondialdehyde (MDA) accumulation. Notably, transient decreases in TBARS values were observed in RD10 (10% starch substitution, 0.425 mm) and RD40 (40% substitution, 0.425 mm) after Day 18 (Figure 2a). The transient decrease in TBARS values is due to MDA reacting with amino acids/peptides in the sausage matrix to form stable Schiff bases, reducing measurable MDA [22].

Figure 2(a, b) The TBARS value of sausages with tomato peel powder during storage.

**Figure:**
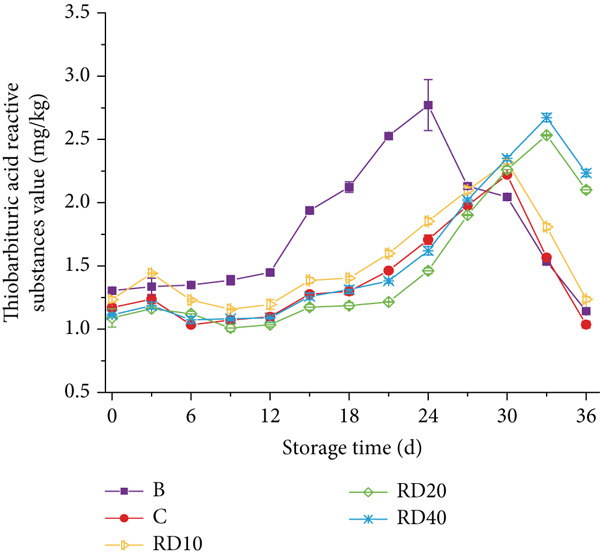
(a)

**Figure:**
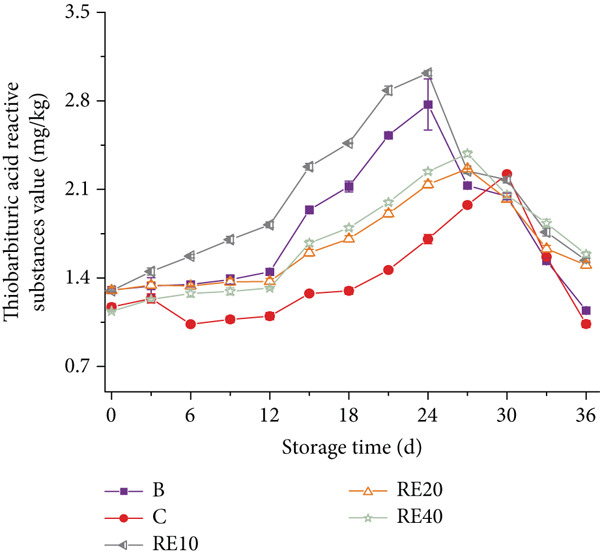
(b)

According to Wang et al. [19], rancidity in meat becomes detectable at a TBARS value of 0.5 mg MDA/kg, while the National Food Safety Standard [18] sets a permissible limit of 2.5 mg/kg. The blank control (Group B) exceeded this threshold on Day 21 (2.81 ± 0.12 mg/kg), whereas TPP‐supplemented groups showed extended shelf life: RD10 and RD40 reached the limit on Day 33 (2.58 ± 0.09 and 2.63 ± 0.11 mg/kg, respectively), indicating a 12‐day delay compared to Group B. This antioxidant effect is mediated by TPP′s bioactive compounds, including lycopene (3–4 mg/100 g) and polyphenols, which scavenge free radicals and chelate prooxidative metals (e.g., Fe^2+^) [7, 13]. Importantly, RD10 outperformed the BHT‐treated group (Group C), which exceeded the limit on Day 30 (2.55 ± 0.08 mg/kg), confirming TPP′s superiority to synthetic antioxidants.

In contrast, RE groups (0.18 mm particle size) showed weaker efficacy: RE10 (10% substitution) exceeded the 2.5 mg/kg limit on Day 27 (2.53 ± 0.07 mg/kg), 6 days earlier than RD10. This is likely due to the larger specific surface area of fine TPP particles, which accelerates antioxidant depletion during storage, whereas coarse particles (0.425 mm) enable sustained release of bioactive compounds [15].

### 3.5. POV

The POV was found to be influenced by both the concentration and particle size of TPP, as illustrated in Figure 3. The results revealed that an increase in TPP concentration led to a decrease in POVs. POV decreases are likely due to peroxide decomposition into secondary oxidation products. (e.g., aldehydes) [20]. Notably, Group B (blank control) reached its maximum POV at 24 days of storage, whereas samples RD10 (10% starch substitution, 0.425 mm), RD20 (20% substitution, 0.425 mm), and RD40 (40% substitution, 0.425 mm) peaked at 30, 33, and 33 days, respectively—indicating a delay of 6–9 days compared to Group B (refer to Figure 3a). According to research by Kurt and Zorba [21], there exists a strong positive correlation between lipolysis and peroxide levels, suggesting that lower POVs are indicative of reduced lipolysis. In our study, it was observed that sausages incorporating TPP with a particle size of 0.425 mm (RD20 and RD40 groups) exhibited a significant delay in the escalation of POVs compared to the BHT‐treated control group (C), achieving a delay period of 3 days. This observation highlights the potent antioxidant capacity of coarse TPP within these formulations.

Figure 3(a, b) The peroxide value (POV) of sausages with tomato peel powder during storage.

**Figure:**
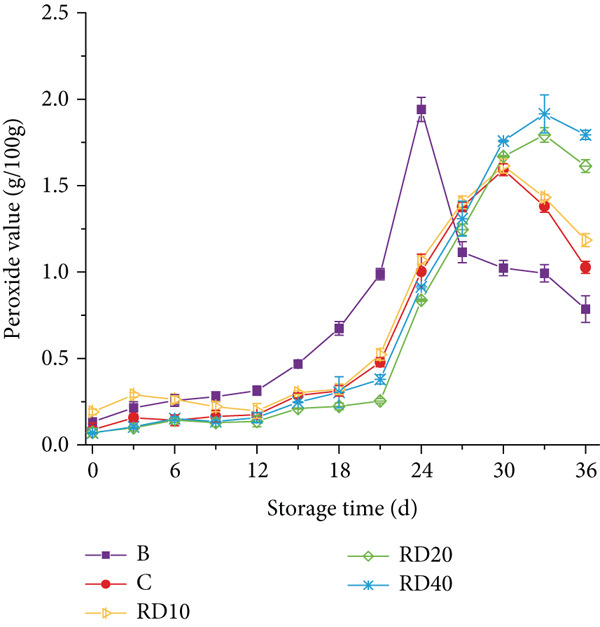
(a)

**Figure:**
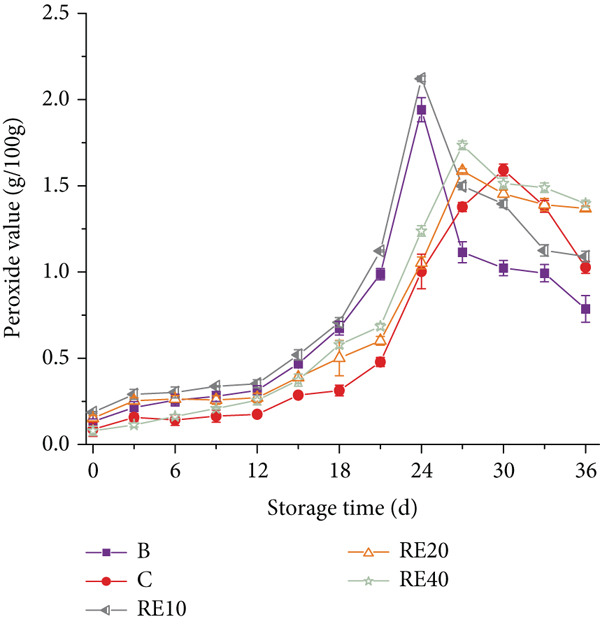
(b)

However, a contrasting trend emerged for smaller particle sizes. As depicted in Figure 3b, the inclusion of TPP with finer particles (RE10 group, 10% substitution, 0.18 mm) did not notably retard the rise in POVs. Furthermore, RE20 (20% substitution, 0.18 mm) and RE40 (40% substitution, 0.18 mm) samples reached their peak peroxide levels after 27 days of storage, which is 3 days earlier than Group C, suggesting diminished antioxidant performance under these conditions. This investigation underscores the importance of particle size optimization in harnessing the full potential of TPP as an antioxidant additive in sausage formulations.

### 3.6. Determination of Fatty Acid Profile and Induction Period

**Figure 4:**
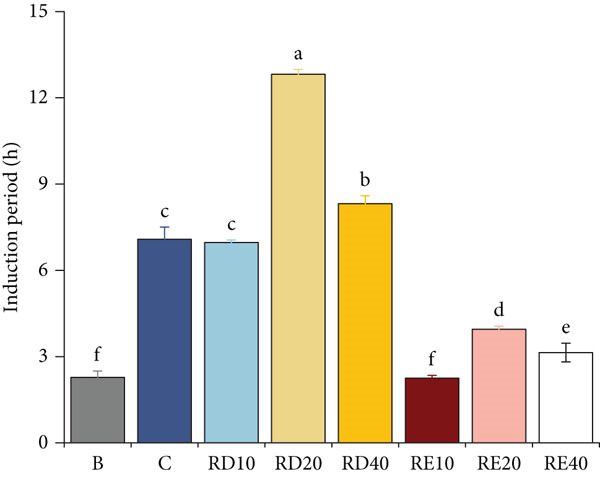
The induction period of sausages with tomato peel powder during storage. Different letters indicate significant differences (Duncan′s test, *p* < 0.05).

In the study, sausages were treated at a high temperature of 120°C to assess the oxidative induction period across various sausage groups, with results depicted in Figure 4. Notably, the RD20 group (20% starch substitution, 0.425 mm particle size) exhibited the most extended induction period, followed by RD40 (40% substitution, 0.425 mm), which was significantly higher than that of Groups B (blank control) and C (0.01% BHT) (*p* < 0.05). Additionally, RE20 (20% substitution, 0.18 mm) and RE40 (40% substitution, 0.18 mm) demonstrated an induction period exceeding that of Group B but significantly lower compared to Group C (*p* < 0.05). The length of the oxidation induction period is inversely proportional to the efficacy of antioxidants; hence, a longer period signifies stronger antioxidant effects. The findings indicate that sausages incorporating tomato particles of 0.425 mm (RD groups) exhibit superior antioxidant properties compared to those with a particle size of 0.18 mm (RE groups). This observation aligns with the POV and TBARS measurements of the sausages. Among the tested samples, RD20 and RD40, particularly RD20, displayed the longest time to reach the peroxide threshold and thus, showed the strongest antioxidant capacity.

### 3.7. Fatty Acids

The incorporation of TPP in the products resulted in significant variations in their fatty acid profiles, as shown in Table 4. The findings revealed that the utilization of this powder led to a substantial augmentation in levels of myristic acid (C14:0), methyl linoleate (C18:1n9t), octadecatrienoic acid (C18:3n3), saturated fatty acids (SFA), and monounsaturated fatty acids (MUFAs), except for sample RE20 (20% starch substitution, 0.18 mm particle size). Notably, sausages processed with a tomato particle size of 0.425 mm (RD groups) displayed an increased presence of arachidonic acid (C20:1 and C20:2). Moreover, compared to Groups B (blank control) and C (0.01% BHT), samples RD10 (10% substitution, 0.425 mm), RD20 (20% substitution, 0.425 mm), RD40 (40% substitution, 0.425 mm), RE10 (10% substitution, 0.18 mm), and RE40 (40% substitution, 0.18 mm) showed a decrease in the concentration of C18:1n9t (*trans* oleic acid). However, there was no statistically significant difference observed in the content of unsaturated fatty acids (UFAs) across all groups, including B, C, RD, and RE.

**Table 4:** Fatty acid profiles (grams per 100 g total fatty acids) and nutritional indices of sausages produced with eight formulations after a 24‐day storage.

**Fatty acid**	**Storage (days)**	**Formulations**							
		**B**	**C**	**RD10**	**RD20**	**RD40**	**RE10**	**RE20**	**RE40**
C14:0	0	0.44 ± 0.01^z^	0.44 ± 0.01^z^	0.44 ± 0.01^z^	0.44 ± 0.01^z^	0.44 ± 0.01^y^	0.44 ± 0.01^z^	0.44 ± 0.01^z^	0.44 ± 0.01^z^
	24	2.18 ± 0.01^fy^	2.19 ± 0.00^fx^	6.84 ± 0.00^aw^	6.67 ± 0.01^bw^	6.19 ± 0.00^ew^	6.44 ± 0.01^cw^	2.09 ± 0.01^gy^	6.37 ± 0.01^dw^
C16:1	0	1.88 ± 0.00^y^	1.88 ± 0.00^y^	1.88 ± 0.00^z^	1.88 ± 0.00^y^	1.88 ± 0.00^y^	1.88 ± 0.00^y^	1.88 ± 0.00^y^	1.88 ± 0.00^y^
	24	0.59 ± 0.10^ez^	0.60 ± 0.00^ez^	1.94 ± 0.00^ay^	1.86 ± 0.01^bz^	1.68 ± 0.01^dz^	1.77 ± 0.01^cz^	0.59 ± 0.01^ez^	1.76 ± 0.01^cz^
C18:1n9t	0	0.16 ± 0.00	0.16 ± 0.00	0.16 ± 0.00	0.16 ± 0.00^y^	0.16 ± 0.00^y^	0.16 ± 0.00^y^	0.16 ± 0.00^y^	0.16 ± 0.00^y^
	24	—	—	—	62.56 ± 0.01^dx^	64.89 ± 0.01^aw^	64.05 ± 0.01^cw^	21.37 ± 0.01^ex^	64.61 ± 0.01^bw^
C18:1n9c	0	37.10 ± 0.00	37.10 ± 0.00	37.10 ± 0.00	37.10 ± 0.00	37.10 ± 0.00	37.10 ± 0.00	37.10 ± 0.00	37.10 ± 0.00
	24	22.01 ± 0.00^b^	21.66 ± 0.01^c^	62.41 ± 0.01^a^	—	—	—	—	—
C18:2n6t	0	0.06 ± 0.00^y^	0.06 ± 0.00^z^	0.06 ± 0.00^z^	0.06 ± 0.00^z^	0.06 ± 0.00^z^	0.06 ± 0.00^z^	0.06 ± 0.00^z^	0.06 ± 0.00^z^
	24	68.44 ± 0.01^cw^	68.80 ± 0.01^bw^	7.91 ± 0.01^hx^	9.00 ± 0.01^dx^	8.43 ± 0.00^ex^	8.22 ± 0.00^gw^	69.56 ± 0.00^aw^	8.41 ± 0.01^fx^
C18:3n3	0	0.86 ± 0.00^y^	0.86 ± 0.00^y^	0.86 ± 0.00^y^	0.86 ± 0.00^y^	0.86 ± 0.00^y^	0.86 ± 0.00^y^	0.86 ± 0.00^y^	0.86 ± 0.00^y^
	24	1.39 ± 0.00^gx^	1.41 ± 0.01^fx^	4.31 ± 0.01^ax^	4.09 ± 0.01^bx^	3.82 ± 0.01^ex^	4.08 ± 0.01^cw^	1.32 ± 0.00^hx^	3.95 ± 0.01^dw^
C20:1	0	0.76 ± 0.00^y^	0.76 ± 0.00^y^	0.76 ± 0.00^y^	0.76 ± 0.00^y^	0.76 ± 0.00^y^	0.76 ± 0.00^z^	0.76 ± 0.00^y^	0.76 ± 0.00^y^
	24	1.49 ± 0.00^fx^	1.49 ± 0.01^fx^	4.74 ± 0.01^aw^	4.42 ± 0.01^bw^	4.21 ± 0.00^dw^	4.28 ± 0.01^cw^	1.39 ± 0.00^gx^	4.12 ± 0.00^ew^
C20:2	0	0.63 ± 0.01^y^	0.63 ± 0.01^y^	0.63 ± 0.01^y^	0.63 ± 0.01^y^	0.63 ± 0.01^x^	0.63 ± 0.01^z^	0.63 ± 0.01^y^	0.63 ± 0.01^y^
	24	1.21 ± 0.00^fx^	1.24 ± 0.00^ex^	3.76 ± 0.02^aw^	3.52 ± 0.01^bw^	3.38 ± 0.01^dw^	3.52 ± 0.01^bw^	1.16 ± 0.01^gx^	3.41 ± 0.00^ew^
∑PUFA	0	16.03 ± 0.02^y^	16.03 ± 0.02^y^	16.03 ± 0.02^y^	16.03 ± 0.02^y^	16.03 ± 0.02^x^	16.03 ± 0.02^x^	16.03 ± 0.02^y^	16.03 ± 0.02^x^
	24	71.04 ± 0.01^cw^	71.44 ± 0.01^bw^	15.98 ± 0.00^ez^	16.61 ± 0.00^dx^	15.62 ± 0.00^hy^	15.81 ± 0.01^fy^	72.03 ± 0.00^aw^	15.77 ± 0.01^gy^
∑UFA	0	55.93 ± 0.03^y^	55.93 ± 0.03^z^	55.93 ± 0.03^y^	55.93 ± 0.03^z^	55.93 ± 0.03^y^	55.93 ± 0.03^y^	55.93 ± 0.03^y^	55.93 ± 0.03^y^
	24	95.13 ± 0.09^cw^	95.19 ± 0.00^bw^	85.07 ± 0.02^hw^	85.45 ± 0.02^gx^	86.40 ± 0.01^dx^	85.91 ± 0.02^fw^	95.37 ± 0.01^aw^	86.25 ± 0.02^ew^
∑SFA	0	2.85 ± 0.02^y^	2.85 ± 0.02^y^	2.85 ± 0.02^y^	2.85 ± 0.02^y^	2.85 ± 0.02^y^	2.85 ± 0.02^z^	2.85 ± 0.02^z^	2.85 ± 0.02^z^
	24	2.18 ± 0.01^fy^	2.19 ± 0.00^fx^	6.83 ± 0.00^ax^	6.67 ± 0.01^bw^	6.19 ± 0.00^ew^	6.44 ± 0.01^cx^	2.09 ± 0.01^gy^	6.35 ± 0.01^dx^
∑MUFA	0	39.91 ± 0.01^x^	39.91 ± 0.01^x^	39.91 ± 0.01^x^	39.91 ± 0.01^y^	39.91 ± 0.01^y^	39.91 ± 0.01^y^	39.91 ± 0.01^w^	39.91 ± 0.01^x^
	24	24.09 ± 0.10^fz^	23.75 ± 0.01^gz^	69.09 ± 0.02^dw^	68.84 ± 0.02^ex^	70.78 ± 0.01^ax^	70.09 ± 0.01^cw^	23.34 ± 0.01^hy^	70.49 ± 0.01^bw^

*Note:* Different letters indicated that there were significant differences among different groups in each metabolite category (Duncan′s test, *p* < 0.05).

These observed fatty acid modifications are closely linked to the antioxidant properties of TPP, which exert dual regulatory effects on lipid stability. Specifically, TPP′s bioactive components—lycopene (3–4 mg/100 g) and polyphenols—scavenge peroxyl radicals (ROO•), thereby reducing oxidative breakdown of PUFAs (e.g., C18:3n3); this mechanism explains the higher UFA retention in RD10/RD20 compared to B/C groups [7, 13]. Concurrently, TPP antioxidants inhibit the isomerization of *cis*‐UFAs (e.g., C18:1n9c) to *trans* forms (e.g., C18:1n9t), which aligns with the lower C18:1n9t levels detected in RD/RE groups relative to B/C [23].

## 4. Conclusion

This research demonstrated that incorporating TPP into sausages enhances flavor and antioxidant capacity, with optimal performance observed at a particle size of 0.425 mm. Processed sausages exhibited decreased lightness (*L* ^∗^) and increased redness (*a* ^∗^) and yellowness (*b* ^∗^), indicating darker, reddish‐yellow coloration. Importantly, TPP with 0.425 mm particle size (RD groups) showed superior antioxidant activity, delaying lipid oxidation (TBARS and POV) and extending shelf life compared to finer particles (0.18 mm, RE groups) and synthetic antioxidant BHT. These findings confirm TPP′s potential as a natural antioxidant and functional ingredient, supporting its use in processed meats to improve nutritional quality and flavor without synthetic additives.

## Ethics Statement

The study does not involve research on human participants and/or animals.

## Conflicts of Interest

The authors declare no conflicts of interest.

## Funding

This study was funded by the Natural Science Foundation of Chongqing Municipality (10.13039/501100005230) (cstc2021jcyj‐msxmX0362).

## Data Availability

The data that support the findings of this study are available from the corresponding author upon reasonable request.
